# A cross-sectional observational study comparing individuals with a symptomatic full-thickness rotator cuff tear with age-matched controls

**DOI:** 10.1016/j.jseint.2023.10.006

**Published:** 2023-11-21

**Authors:** Marianne Roos, Michaël Bertrand-Charette, Marc-Olivier Dubé, Jean Tittley, Mélanie Brisson, Luc Chau, Jackie L. Whittaker, François Desmeules, Catherine Mercier, Jean-Sébastien Roy

**Affiliations:** a Faculty of Medicine, School of Rehabilitation Sciences, Université Laval, Québec, QC, Canada; b Centre interdisciplinaire de recherche en réadaptation et intégration sociale (Cirris), Québec, QC, Canada; c Centre Hospitalier Universitaire (CHU) de Québec, Québec, QC, Canada; d Radiologie Mailloux, Québec, QC, Canada; e Department of Physical Therapy, University of British Columbia, Vancouver, BC, Canada; f Arthritis Research Canada, Vancouver, BC, Canada; g Faculty of Medicine, School of Rehabilitation, Université de Montréal, Montreal, QC, Canada; h Orthopaedic Clinical Research Unit, Centre de recherche de l’Hôpital Maisonneuve-Rosemont (CRHMR), CIUSSS de l’Est-de-l’Île de Montréal, Montreal, QC, Canada

**Keywords:** Anatomical, Biomechanical, Neuromuscular, Pain sensitivity, Psychosocial, Sociodemographics

## Abstract

**Background:**

A full-thickness rotator cuff tear (FTRCT) is defined as a complete tear of one of the four rotator cuff muscle tendons (supraspinatus, infraspinatus, subscapularis or teres minor). This condition can lead to pain and reduced function. However, not all FTRCT are symptomatic. A better understanding of the characteristics that lead some individuals with FTRCT to experience pain is fundamental to improve strategies used to manage this condition. This level II descriptive study aimed to explore potential sociodemographic, anatomical, psychosocial, pain sensitivity, biomechanical and neuromuscular variables that may differ between individuals with symptomatic FTRCT and age-matched individuals with asymptomatic shoulders.

**Methods:**

In this cross-sectional observational study, adults aged 50 to 80 years of age, either with symptomatic FTRCT or no shoulder pain, were recruited via convenience sampling. Participants filled out questionnaires on sociodemographic and psychosocial variables. Then, various tests were performed, including pain pressure threshold, shoulder range of motion, shoulder muscle strength, shoulder ultrasound and radiologic examination, and sensorimotor functions testing. Each variable was compared between groups using univariate analyses (independent *t*-tests, Mann-Whitney U tests, exact probability Fisher tests). Significance was set at 0.05.

**Results:**

FTRCT (n = 30) and Control (n = 30) groups were comparable in terms of sex, age, and number of comorbidities. The symptomatic FTRCT group showed a higher proportion of smokers (*P* = .026) and more participants indicated consuming alcohol or drugs more than they meant to (*P* = .010). The FTRCT group had a significantly higher prevalence of glenohumeral osteophytes (48% vs. 17%; *P* = .012). Participants in the FTRCT group were significantly more stressed (*P* = .04), anxious (*P* = .003) and depressed (*P* = .002). The FTRCT group also showed significantly higher levels of pain catastrophisation (*P* < .001) and sleep disturbance (*P* < .001). The FTRCT group showed significantly lower range of motion for flexion (*P* < .001), and external rotation at 0° (*P* < .001) and 90° (*P* < .001) of abduction. Isometric strength in both abduction and external rotation were weaker (*P* = .005) for the FTRCT group.

**Conclusion:**

Sociodemographic, anatomical, psychosocial and biomechanical variables showed statistically significant differences between the FTRCT and Control groups.

Shoulder pain is the third most prevalent site of musculoskeletal (MSK) pain,^51^^,^^90^ and about 4% of adults visit a physician for shoulder pain per year.^26^ As the population ages and older workers make up an increasing portion of the workforce,^4^ the burden of shoulder pain and its significant negative impact on health-related quality of life and work-related disability is growing.^13^ Rotator cuff (RC) related shoulder pain accounts for 50% to 85% of diagnoses for shoulder pain.^77^ These disorders include a range of diagnostic labels such as subacromial impingement, RC tendinopathy, partial or full-thickness tear, long head of the biceps tendinosis, and subacromial bursitis. These conditions are associated with pain-related disabilities that increase with age.^13^^,^^27^^,^^32^

The most serious RC-related shoulder pain, from an anatomical and structural standpoint, is a full-thickness rotator cuff tear (FTRCT). A FTRCT is defined as a tear of at least one of the four RC muscles (supraspinatus, infraspinatus, subscapularis or teres minor) where the tear goes through the entire thickness of the tendon.^40^ It is estimated that nearly 20% of the population presents some level of RC tear,^88^ with an incidence that increases with age.^27^ In many cases, symptomatic FTRCT has severe debilitating effects, resulting in decreased ability to execute activities of daily living, and in decreased quality of life and general health status.^55^^,^^66^ Symptomatic FTRCT are common among workers and can contribute to significant work absence, early retirement and the need for social support.^69^

On the other hand, FTRCT is commonly seen in asymptomatic individuals, as approximately two-thirds of people with FTRCT on medical imaging are asymptomatic.^27^^,^^66^ In fact, the association between the presence of FTRCT observed on medical imaging and pain/pain-related disability is poor,^22^^,^^56^^,^^57^ as significant structural abnormalities of the shoulder tissues are often seen in people without any shoulder pain.^22^^,^^37^^,^^56^^,^^57^ Thus, the presence of a FTRCT alone cannot explain the level of pain and disability reported by people with symptomatic FTRCT. The etiology of pain in individuals with symptomatic FTRCT is still poorly understood and is likely multifactorial.^30^ It could be explained by personal, occupational, anatomical, biomechanical, or psychosocial factors. A better understanding of FTRCT pain is fundamental to the development of effective strategies to overcome the burden of these disorders.

The objective of this study was to explore the sociodemographic, anatomical, psychosocial, pain sensitivity, biomechanical and neuromuscular variables that may differ between individuals with symptomatic degenerative FTRCT and age-matched individuals with asymptomatic shoulders (with and without asymptomatic FTRCT).

## Materials and methods

This is a descriptive study of cross-sectional design that follows the Strengthening the Reporting of Observational Studies in Epidemiology reporting guideline. Ethical approval was obtained from the sectorial rehabilitation and social integration research ethics committee of the CIUSSS-CN (#2017-565) and participants provided written consent.

### Participants

Participants were adults aged 50 to 80 years,^78^ either with symptomatic FTRCT or no shoulder pain, recruited via convenience sampling. Adults with symptomatic FTRCT were recruited at a diagnostic imaging clinic in Quebec City, Canada, by verbally inviting individuals already scheduled for a shoulder ultrasound (US) examination and who tested positive for a FTRCT, to participate. Asymptomatic age-matched individuals were recruited through the electronic mailing list of current and retired employees of *Université Laval* and senior organizations such as *Fédération de l'Âge d'Or du Québec*. Participants in the control group were with or without FTRCT (confirmed during an US examination). We initially aimed to recruit a balanced group of age-matched individuals with and without asymptomatic FTRCT. Recruiting individuals with asymptomatic FTRCT, however, proved to be extremely difficult, so we ended up including mostly asymptomatic individuals without FTRCT.

Inclusion criteria: Participants were assigned to the Symptomatic FTRCT Group if they: 1) had a US radiologist-confirmed FTRCT (all RC tendons were assessed); 2) reported shoulder pain, at rest or during movement, of at least 2/10 on a numerical pain rating scale evaluating usual shoulder pain; 3) responded positively to ‘In the past four weeks, have you had pain in your shoulder’ and ‘If yes, was this pain bad enough to limit your usual activities or change your daily routine for more than one day?’.^15^ Participants were assigned to the Control Group if they: 1) reported no current shoulder pain (0/10 on a numerical pain rating scale evaluating usual shoulder pain); 2) responded negatively to: ‘In the past four weeks, have you had pain in your shoulder’; 3) did not report any history of past significant shoulder pain (pain was considered significant if its intensity was greater than or equal to 2/10 for longer than six weeks and prompted either the use of medication or a medical consultation).^35^ Exclusion criteria for all participants: 1) unable to understand French or English; 2) history of upper limb fracture; 3) previous shoulder surgery; 4) cervicobrachialgia or shoulder pain reproduced by neck movement; 5) shoulder capsulitis; 6) rheumatoid, inflammatory or neurological disease; 7) corticosteroid injection in the previous six weeks; and 8) cognitive problems interfering with evaluations (Mini-Mental State Examination ≥ 24).^11^

### Procedures

For symptomatic participants, the first evaluation took place at a diagnostic imaging clinic, where all radiologists involved in the project have at least 10 years of experience in MSK imaging. Radiologists evaluated their scheduled shoulder patients with US imaging and, when a FTRCT was discovered, asked the patient whether they would be interested in participating in the research project. If the answer was positive, the radiologist introduced the patient to the on-site member of the research team, who described the project and invited them to participate. Those who were interested provided informed consent before undergoing eligibility screening with the same PT, followed by an X-ray evaluation (shoulder anteroposterior and lateral views). Within a month, included participants took part in a second evaluation at the *Centre interdisciplinaire de recherche en réadaptation et en intégration sociale* (Cirris) conducted by a PT. The second evaluation comprised questionnaires on sociodemographic variables and psychosocial factors. Then, various tests were performed, including the processing of nociceptive information, shoulder range of motion, shoulder muscle strength, AHD (using US imaging), and upper limb sensorimotor functions testing. For asymptomatic participants, this evaluation (at the Cirris) was preceded by provision of informed consent on site before commencing the questionnaires and tests. Then, all asymptomatic participants were scheduled for US and X-ray evaluations of their two shoulders at the diagnostic imaging clinic.

### Outcome measures

Several outcomes were chosen based on studies that have looked at the occurrence and persistence (chronicity) of shoulder pain and of other types of MSK disorders.^1^^,^^10^^,^^18^^,^^33^^,^^46^^,^^60^^,^^64^^,^^68^^,^^81^^,^^87^ They are presented in categories. 1) Sociodemographics: education level, smoking status, alcohol and drug consumption, comorbidities (using the validated and reliable [ICC Intraclass correlation coefficient (ICC) = 0.91] Self-Administered Comorbidity questionnaire^34^^,^^70^). Physical demands of their sport/work were evaluated with the *Revised Upper Extremity Work Demands (UEWD-R) Scale* for current and previous occupations when applicable.^6^ The *UEWD-R* previously showed good validity and test-retest reliability (ICC = 0.79) when used to evaluate workload in the upper extremities.^6^ 2) Anatomical: RC and shoulder joint structural integrity (RC tendons, acromioclavicular joints, presence of bursitis and/or osteophytes) were determined with a US examination and X-rays performed by a radiologist.^14^ FTRCT were classified as small (<10 mm), medium (10 to 30 mm) or large (>30 mm).^67^ 3) Psychosocial: Psychosocial measures were selected to assess constructs commonly associated with poorer status or outcomes for MSK and shoulder pain.^9^^,^^23^^,^^36^ They include the 4-item Perceived Stress Scale (PSS), an instrument with adequate reliability (*r* = 0.76)^80^ which aims to measure how different situations affect one’s feelings and perceived stress,^44^ the Pain Catastrophizing Scale,^21^^,^^75^ which reliably (ICC = 0.83-0.93)^85^ measures the extent to which individuals have overly negative and pervasive thoughts about pain, the Patient Health Questionnaire (PHQ), a reliable (ICC = 0.84-0.94)^91^ tool developed to diagnose the presence and severity of depression,^74^ the State-Trait Anxiety Inventory, a measure of state and trait anxiety,^2^ and the PROMIS short-form 4a, a measure of sleep disturbance with moderate reliability (ICC = 0.62-0.71).^7^ 4) Pain sensitivity: Pressure pain threshold (PPT), which evaluates modulation of pain-related processes in the central nervous system,^47^ was assessed. PPT is defined as the minimal amount of pressure at which pain is perceived. To discriminate between peripheral and central sensitization, asymptomatic sites, distant to the site of pain, were included.^64^ Therefore, PPT was assessed at the middle deltoid on the painful/FTRCT side and the middle deltoid and tibialis anterior muscles on the opposite side, or at the middle deltoid on both sides and tibialis anterior muscles of the dominant side, for the symptomatic and asymptomatic groups, respectively, using a calibrated mechanical pressure algometer at an applied rate of 0.5 kg/cm^2^ per second.^64^ Pressure at which pain was perceived was recorded (mean of three trials).^64^ Intrarater reliability (ICC) of PPT of the middle deltoid was shown to be 0.86-0.96.^82^ 5) Biomechanical: The biomechanical assessment included a physical examination of the painful shoulder in the symptomatic FTRCT group and of one of the shoulders in the control group (paired with the FTRCT group), with measurements of active shoulder range of motion [ROM] (flexion, external rotation at 0°, and internal and external rotation at 90° of abduction) using a goniometer or inclinometer (intrarater ICC = 0.94-0.98),^31^ isometric strength testing including humeral abductors and external rotators (intrarater ICC = 0.85-0.96)^8^ and grip strength (intrarater ICC = 0.93-0.96)^29^ (MedUp handheld dynamometer^3^ [MedUp, Tokyo, Japan] and Jamar hydraulic hand dynamometer [Greendale, WI, USA] respectively). 6) Neuromuscular: Shoulder sensorimotor function was estimated using acromiohumeral distance (AHD) measures and sensorimotor upper limb functions were assessed using a bilateral Kinarm Exoskeleton Lab (Kinarm, Kingston, ON, Canada), a robotic device that allows precise kinematic measurement during combined movements of the shoulder (horizontal abduction-adduction) and elbow (flexion-extension) joints in the horizontal plane. This device also includes a 2D virtual environment allowing the presentation of targets and visual feedback. Participants performed three standard Kinarm tasks: Arm Position Matching (a 3-minute proprioceptive task in which the robot moves one of the subject’s arms to a given position, and the subject is instructed to move their other arm to the mirror-image position without visual feedback), Ball On Bar (a 3-minute inter-limb coordination task in which participants have to move a virtual ball onto each presented target as quickly and accurately as possible) and Object Hit (a 2-minute accuracy, rapidity and spatial awareness task in which participants use virtual paddles to hit and push away balls that appear randomly from various locations across the top of the screen). The Kinarm tasks produce percentile scores compared to normative data; the lower the percentile, the better the performance. The reliability of these tasks has been demonstrated.^17^^,^^48^^,^^79^ AHD was measured using an US scanner (Logic E9; GE Healthcare, Milwaukee, WI, USA) and was defined as the tangential distance between the hyperechoic bony landmarks of the humeral head and the inferior edge of the acromion.^14^ AHD measurements were taken with the arm at rest and 60° of active abduction using the procedure described by McCreesh et al.^53^ Several groups, including ours, have shown that these US measures are reliable with the Logic E9 (ICC = 0.98; MDC = 0.7 mm).^53^^,^^71^

### Statistical analysis

Sample size calculation (G∗Power 3.1.9; α of 0.05; β of 0.2) was established for one of our variables, the AHD at 60° of abduction (cm). A previous validity study reported a minimal detectable change value of 0.07 cm.^53^ To detect a mean difference of 0.07 cm assuming a common standard deviation (0.10 cm),^16^^,^^19^ a sample of 30 participants per group was needed (two-sided test, 1-β = 0.8, α = 0.05) to have sufficient statistical power to detect significant between-group differences. Sample size was also calculated for other single variables such as ROM and isometric strength. For each of these variables, the number of participants suggested was below the sample size required for AHD. Therefore, the sample size was based on the AHD as presented above. Since this was an exploratory study, the sample size did not consider multivariate analysis or multiple comparisons and was calculated for a single variable between-group comparison test.

The study sample was characterized using descriptive statistics for each group. All variables were compared between groups using independent *t*-tests or Mann-Whitney U tests (continuous variables) and exact probability Fisher tests (categorical variables). Significance was set at 0.05.

## Results

Both groups were comparable in terms of sex, gender, age, height, weight and dominance (Table I).

**Table I:** Participants’ characteristics and sociodemographic variables.

	FTRCT group (n = 30)	Control group (n = 30)	*P* value
Sex (Male/Female)	18/12	14/16	.400
Age	65.9 (6.2)	65.2 (8.7)	.708
Height	164.9 (8.1)	164.9 (8.0)	.988
Weight	81.3 (15.4)	77.1 (17.2)	.317
Dominance (Right/Left)	27/3	28/2	.640
Dominant side affected (Yes/No)	20/10	-	-
Level of Education			<.001
Diploma below bachelor’s degree	25	12	
At least a bachelor’s degree	5	18	
Incomes			.879
<50,000$	10	8	
50,000$-100,000$	8	9	
>100,000$	11	10	
Smoking			.026
Never smoked	8	18	
Current smoker	1	0	
Used to smoke	21	12	
Consumed alcohol or used drugs more than meant to			.010
Never	14	25	
Rarely	10	3	
Sometimes	6	1	
Often	0	1	
Felt wanted or needed to cut down on drinking or drug use			.223
Never	22	26	
Rarely	3	3	
Sometimes	5	1	
Often	0	0	

*FTRCT*, full-thickness rotator cuff tear.

### Sociodemographics

Symptomatic FTRCT and Control groups were comparable in terms of number of comorbidities. Regarding the level of education, 83% of participants in the FTRCT group had an education level below a bachelor’s degree, compared to 40% in the Control group (*P* < .001), even though incomes were similar across groups (*P* = .879; Table I). The FTRCT group also showed a higher proportion of smokers (*P* = .026) and more of its participants indicated consuming alcohol or drugs more than they meant to (Table I; *P* = .010). When comparing the two groups with regards to physical work/sport demands, participants in the FTRCT group previously had higher upper extremity physical demands than participants in the Control group (*P* = .008; Table II). However, current physical work demands were similar between groups (*P* = .256).

**Table II:** Anatomical, psychosocial, pain sensitivity, biomechanical and neuromuscular variables.

	FTRCT group (n = 30)	Control group (n = 30)	Mean difference [95%, CI]	*P*
Upper extremity work/Sport physical demands (revised upper extremity work demands Scale)				
Current	10.7 (4.0)	10.1 (3.0)	0.6 [−1.2, 2.4]	.256
Previous	13.8 (5.8)	10.7 (3.7)	3.1 [0.6, 5.6]	.008
Questionnaires				
Perceived stress scale (0-16)	4.8 (3.6)	2.5 (2.7)	2.2 [0.6, 3.9]	.04
State-trait anxiety inventory (20- 80)	37.0 (12.1)	29.2 (8.7)	7.8 [2.3, 13.2]	.003
Pain catastrophizing scale (0-52)	17.3 (12.5)	7.2 (6.9)	10.1 [4.9, 15.3]	<.001
Patient Health Questionnaire (0-27)	4.6 (4.6)	1.8 (2.4)	2.8 [0.9, 4.6]	.002
PROMIS short-form 4a (sleep disturbance; 4-20)	10.5 (3.3)	7.5 (2.7)	3.1 [1.5, 4.7]	<.001
Pressure pain threshold (N)				
Deltoid – painful side	22.4 (9.8)	24.3 (10.1)	1.9 [−7.1, 3.3]	.238
Deltoid – nonpainful side	25.2 (13.1)	25.0 (10.3)	0.2 [−6.0, 6.3]	.477
Anterior tibialis	38.2 (22.1)	36.2 (15.0)	2.0 [−7.8, 11.8]	.345
Shoulder range of motion (°)				
Flexion	152.7 (15.6)	165.5 (8.4)	−12.8 [−19.3, −6.3]	<.001
External rotation at 0°	60.8 (15.6)	75.3 (9.8)	−14.53 [−21.2, −7.8]	<.001
External rotation at 90°	76.7 (17.6)	93.3 (9.1)	−16.6 [−23.9, −9.3]	<.001
Internal rotation at 90°	59.3 (11.4)	62.3 (11.8)	−3.1 [−9.1, 3.0]	.158
Shoulder isometric strength (Nm)				
Abduction	45.8 (24.9)	64.9 (29.7)	−19.1 [−33.3, −5.0]	.005
External rotation	16.3 (10.4)	23.1 (9.3)	−6.8 [−11.9, −1.7]	.005
Grip strength (N)	49.1 (22.6)	37.3 (16.2)	11.8 [1.7, 21.9]	.011
Acromiohumeral distance (cm)				
At rest	1.05 (0.26)	1.12 (0.18)	−0.08 [−0.20, 0.04]	.093
At 60° of abduction	0.88 (0.24)	0.89 (0.22)	−0.06 [−0.13, 0.11]	.452
Kinarm (%)				
Arm position matching (proprioception)	50.3 (29.7)	52.7 (29.3)	−2.4 [−18.6, 13.7]	.383
Ball On Bar (coordination)	64.4 (28,9)	71.3 (32.8)	−7.0 [−23.6, 9.6]	.201
Object hit (accuracy, rapidity and spatial awareness task)	46.8 (32.8)	41.8 (31.5)	5.0 [−12.6, 22.6]	.286

*FTRCT*, full-thickness rotator cuff tear; *PROMIS*, Patient-Reported Outcomes Measurement Information System; *CI*, confidence interval.

Group data presented as mean (standard deviation).

### Anatomical measurements

US findings show that the supraspinatus was the most frequently torn tendon (>70%) in the FTRCT group. Twenty-six percent of the participants in the FTRCT group had a small tear, 59% a medium tear and 15% a large tear. In the Control group, one participant had a small asymptomatic FTRCT (subscapularis tendon). Finally, more participants in the FTRCT group showed signs of subacromial bursitis (87%) compared to the Control group (47%) (*P* < .001). Regarding radiographic findings, the FTRCT group presented a significantly higher prevalence of glenohumeral osteophytes (48% vs. 17%; *P* = .012) and tended to present a higher prevalence of acromioclavicular osteophytes (88% vs. 76%; *P* = .069), though the difference was not statistically significant.

### Psychosocial outcomes

**Figure 1:**
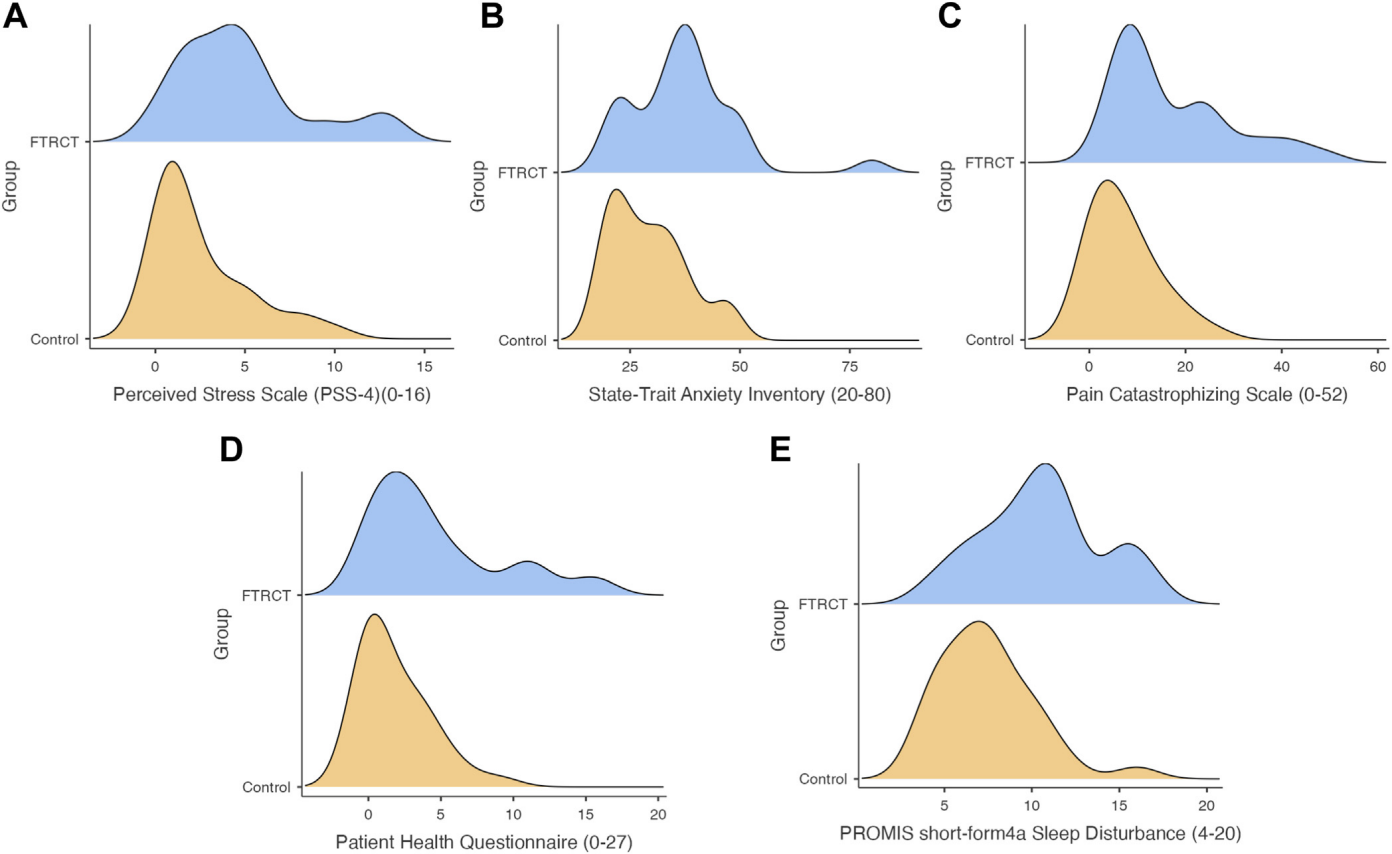
Psychosocial outcomes average frequency curves. (**A**) Perceived Stress Scale (PSS-4); (**B**) Patient Health Questionnaire (PHQ); (**C**) Pain catastrophizing Scale; (**D**) State-Trait Anxiety Inventory; (**E**) PROMIS short-form 4a (Sleep Disturbance).

Participants in the FTRCT group were significantly more stressed (*P* = .04), anxious (*P* = .003) and depressed (*P* = .002) (Table II). The FTRCT group also showed significantly higher levels of pain catastrophization (*P* < .001) and sleep disturbance (*P* < .001) compared to the Control group. (Fig. 1)

### Pain sensitivity

**Figure 2:**
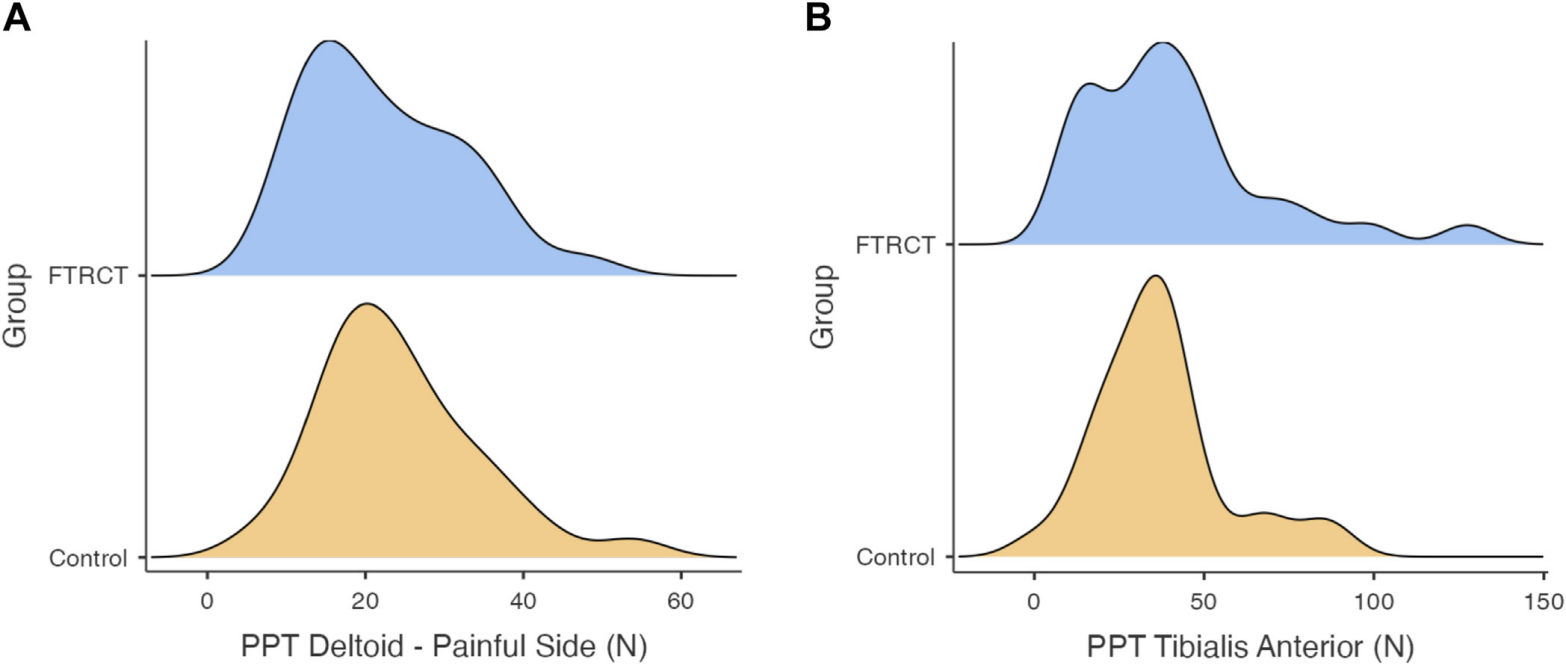
Pain sensitivity average frequency curves. (**A**) Deltoid pain pressure threshold on the symptomatic side; (**B**) Tibialis anterior pain pressure threshold.

PPT was measured bilaterally at the deltoid and at one site on the tibialis anterior. No significant difference was found between the two groups at any site (Table II; Fig. 2).

### Biomechanical outcomes

Regarding shoulder ROM (Table II), the FTRCT group showed significantly lower ROM for flexion (*P* < .001), and external rotation at 0° (*P* < .001) and at 90° (*P* < .001) of abduction. The groups were comparable for internal rotation at 90° of abduction (*P* = .158). Regarding shoulder isometric strength, both abduction and external rotation were weaker (*P* = .005) for the FTRCT group. However, grip strength was stronger for participants in the FTRCT group (Table II; *P* = .011).

### Neuromuscular control

**Figure 3:**
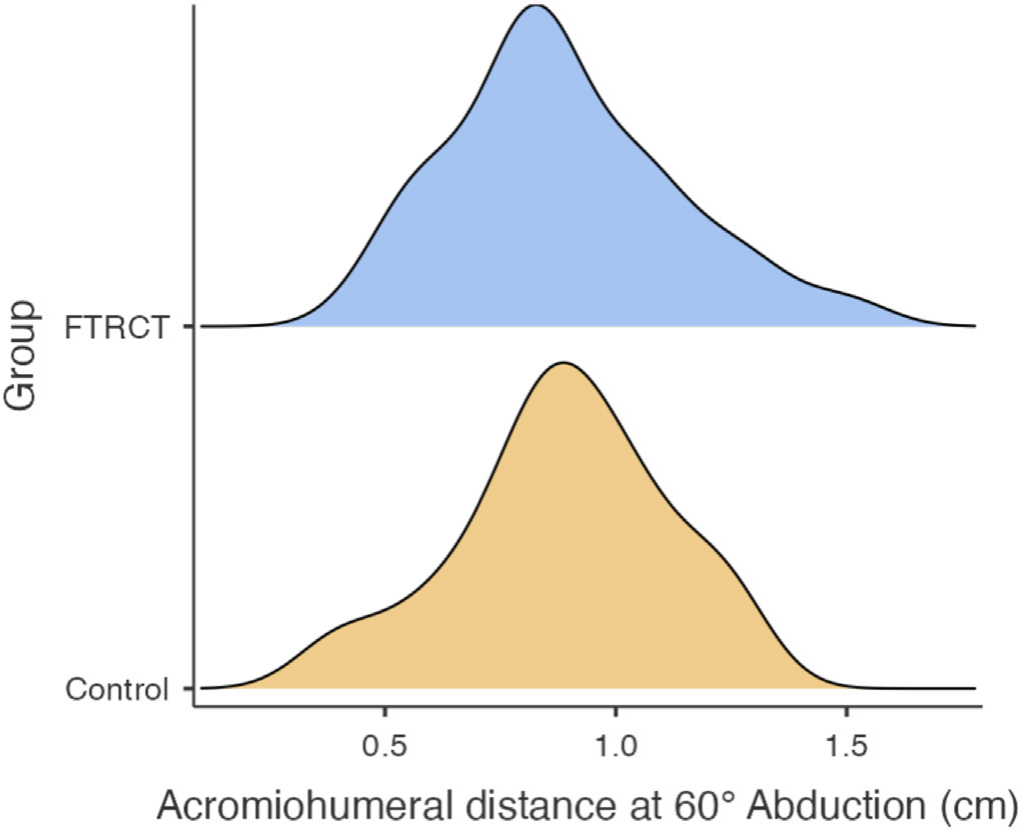
Acromiohumeral distance (cm) at 60° of arm abduction average frequency curves.

When comparing AHD (Table II), the groups had similar results at rest (*P* = .093) and at 60° of abduction (*P* = .452) (Fig. 3). As for upper extremity sensorimotor functions, groups were also similar in terms of position matching and bilateral upper limb sensorimotor performance (Table II).

## Discussion

The objective of this cross-sectional study was to identify variables that may differ between individuals with symptomatic FTRCT and aged-matched individuals with asymptomatic shoulders. By comparing six categories of variables, this study aimed to better understand the factors that may explain the presence of pain in individuals with a US-diagnosed FTRCT.

Participants in the FTRCT group had a lower level of education and described previous higher physical demands at work compared to the control group. One reason for this may be that recruitment through a university mailing list is likely to induce a bias toward less physically demanding jobs. Still, previous studies have suggested that a lower level of education is related to jobs associated with more physically demanding tasks,^12^^,^^83^ and that more physically demanding jobs are linked to a higher incidence of shoulder injuries.^50^^,^^62^^,^^88^ Moreover, participants in the FTRCT group were significantly more likely to be smokers or to consume alcohol or drugs more than they meant to. Both of these variables have previously been identified as risk factors for FTRCT.^1^^,^^5^^,^^63^

The rate of FTRCT in the control group was minimal. Only one participant in the group of 30 (3%) presented an asymptomatic FTRCT, which is much lower than what has been reported (17%-30%) in asymptomatic individuals aged between 50 and 80 year old.^56^^,^^78^ This discrepancy could be explained by the significantly lower prevalence of factors associated with FTRCT in control group participants (eg, higher physical demands at work, smoking). Glenohumeral osteophytes were observed more frequently in the FTRCT group and previous studies have shown that the presence of subacromial osteophytes increases the risk of FTRCT.^38^^,^^65^ However, our results only allow us to highlight a between-group difference without determining whether the latter is a risk factor for symptomatic FTRCT. It is still highly debated as to whether degenerative changes are contributing factors to the evolution of FTRCT or one of its consequences.^45^^,^^61^^,^^89^

Participants in the FTRCT group also showed higher levels of stress, anxiety, depression, pain catastrophizing and sleep disturbance. White et al demonstrated that high PSS (Perceived Stress Scale) scores are associated with higher pain intensity and pain interference levels in older adults.^86^ Anxiety, catastrophization, depression and sleep deprivation are other factors associated with increased pain perception in various pain conditions,^20^^,^^24^^,^^42^^,^^58^ while anxiety, depression and catastrophizing are also associated with pain chronicization.^41^^,^^54^ The poorer psychosocial outcomes observed in the FTRCT group could be related to the level and duration of pain. However, the cross-sectional design used in this study and the characteristics of our control group do not allow us to compare these outcomes between symptomatic and asymptomatic individuals with FTRCT, limiting our ability to conclude.

As expected, symptomatic FTRCT was associated with reduced ROM and isometric strength.^43^^,^^52^ Interestingly, asymptomatic FTRCT is not consistently associated with ROM and strength deficits.^43^ This could in part be due to participants compensating with different muscle activation patterns.^73^ The absence of pain, and therefore of its inhibition of muscle contraction, could also explain why individuals with asymptomatic FTRCT do not exhibit strength deficits.^25^ Interestingly, grip strength was stronger in the FTRCT group. This is not entirely surprising since grip strength was not found to be correlated with FTRCT in a previous study by Manske et al.^49^

When comparing upper limb sensorimotor functions using the Kinarm, no significant difference was found between the two groups. These findings are not consistent with previous studies showing that shoulder proprioception is altered by FTRCT.^28^^,^^76^ Takahashi et al looked at passive joint position sense using the detection of passive motion threshold for abduction and external rotation.^76^ They highlighted significant differences between the affected and unaffected sides as well as between partial to medium, and large to massive tears. Gumina et al assessed active joint position sense at different angles (30°, 60°, 90°, 120° and 150°) of shoulder flexion.^28^ Individuals with FTRCT displayed significantly larger errors in active joint position sense compared to healthy controls for all angles. This discrepancy between our results and the literature could be due to the variability in protocols used for assessing shoulder proprioception across studies and the measurement of different proprioception constructs (eg, joint position sense vs. kinesthesia). The protocol used in this study involved 2-dimensional arm position matching tasks with the arm supported in the Kinarm. In a task against gravity, sense of effort might play a larger role than in a task in which the arm is fully supported. Thus, a 3-dimensional task without any support of the arm might have led to different results.

Both groups also showed similar AHD results at 60° of abduction. Previous literature has suggested that symptomatic FTRCT are associated with superior migration of the humeral head (based on data collected using magnetic resonance imaging and X-rays) which results in a reduced AHD.^59^^,^^72^^,^^84^ However, potential reasons for this disparity are the number of isolated RC tears (only one affected tendon) and smaller tear size. Indeed, almost half of the participants in our sample only had either one affected tendon (44%) or low-to-moderate tear size (47%), and previous research has shown that the magnitude of humeral head migration is correlated with the number of tears as well as their size.^72^

As stated above, sociodemographic, anatomical, psychosocial and biomechanical variables are the four main categories that showed statistically significant differences between the FTRCT and the Control groups. This suggests that FTRCT pain could be explained by biological, psychological and social components, and above all else, by a combination of these factors. First, the presence of symptoms in FTRCT could be associated with actual tissue damage observed on anatomical measurements. It is well known that larger tears are strongly related to the development of symptoms.^43^^,^^52^ On the other hand, there were also significant between-group differences for psychosocial outcomes such as self-reported stress, anxiety, depression, catastrophizing and sleep disturbance. Since all these outcomes have been associated with increased pain perception and may be related to pain intensity and chronicization,^20^^,^^24^^,^^41^^,^^42^^,^^54^^,^^86^ they could contribute to the presence of symptoms following FTRCT. Therefore, it may be important to systematically assess these variables with individuals who present with symptomatic FTRCT. Finally, it should be noted that lower levels of education, history of smoking and amount of alcohol and drug consumption may also be associated with symptomatic FTRCT. This is consistent with the literature as MSK pain has been associated with lifestyle habits.^39^

### Strengths and limitations

This study has limitations. This was a level II descriptive study aimed at generating hypotheses of differences between individuals with symptomatic degenerative FTRCT and age-matched individuals with asymptomatic shoulders. It was, thus not powered to perform multivariate analyses. Second, we acknowledge a potential selection bias in the recruitment of participants through our convenience sampling. However, we believe that this potential bias did not overly influence our results, as recruiting via university mailing lists allows us to reach support staff or family members of university employees with lower levels of education. We also recruited participants through the mailing list of a senior association which includes over 550,000 members of the 50+ years old community. Nonetheless, future studies should include a greater proportion of individuals with a diploma below bachelor’s degree in the control group, or make sure that both groups compared are matched for their level of education. Finally, we cannot exclude the presence of measurement bias. Radiologists were not blinded to whether participants were symptomatic or not, which could have impacted on findings they reported based on US imaging. Also, even though we used standardized procedures with evidence of validity and reliability, US and PPT measurements remain somewhat user dependent.

Strengths of our study include that our groups were similar in terms of sex, gender, height, weight, dominance, and number of comorbidities. Moreover, we included outcomes covering a wide variety of impairments present in this population, thus providing a good overview and a fairly complete representation of FTRCT participants. These outcomes cover different spheres at the biological (biomechanical), psychological and social levels, which ensure a better representation of the factors that may explain the development and/or persistence of pain.

## Conclusion

By comparing different clinical measurements and psychosocial outcomes, this descriptive study identified four categories of variables (sociodemographic, anatomical, psychosocial and biomechanical variables) that may differ between individuals with symptomatic degenerative FTRCT and age-matched individuals with asymptomatic shoulders, which is not surprising considering the multifactorial etiology of MSK pain. Future studies are needed to confirm these between-group differences with multivariate analyses, assess the time course of these variables and explore possible correlations with pain, as well as their preponderance to be a state or a trait in symptomatic individuals.

## Disclaimers:

Funding: This study was funded by the Canadian MSK Rehab Research Network.

Conflicts of interest: MR, MBC and MOD receive a Doctoral Training Scholarship from the *Fonds de Recherche Québec-Santé* (FRQ-S). FD, CM and JSR are supported by salary awards from the FRQ-S. JLW is supported by salary awards from the Arthritis Society and Michael Smith Health Research BC. The other authors, their immediate families, and any research foundation with which they are affiliated have not received any financial payments or other benefits from any commercial entity related to the subject of this article.
